# The Contribution of Foods Prepared Outside the Home to the Diets of 18- to 30-Year-Old Australians: The MYMeals Study

**DOI:** 10.3390/nu13061761

**Published:** 2021-05-21

**Authors:** Lyndal Wellard-Cole, Alyse Davies, Juliana Chen, Jisu Jung, Kim B. Bente, Judy Kay, Wendy L. Watson, Clare Hughes, Anna Rangan, Kalina Yacef, Irena Koprinska, Kathy Chapman, Nim Ting Wong, Luke Gemming, Cliona Ni Mhurchu, Adrian Bauman, Margaret Allman-Farinelli

**Affiliations:** 1 Charles Perkins Centre, Nutrition and Dietetics Group, School of Life and Environmental Sciences, The University of Sydney, Sydney, NSW 2006, Australia; alyse.davies@sydney.edu.au (A.D.); juliana.chen@sydney.edu.au (J.C.); anna.rangan@sydney.edu.au (A.R.); nwon2694@alumni.sydney.edu.au (N.T.W.); luke.gemming@sydney.edu.au (L.G.); margaret.allman-farinelli@sydney.edu.au (M.A.-F.); 2 Cancer Prevention and Advocacy Division, Cancer Council NSW, Sydney, NSW 2011, Australia; wendyw@nswcc.org.au (W.L.W.); clareh@nswcc.org.au (C.H.); 3 School of Computer Science, The University of Sydney, Sydney, NSW 2006, Australia; jjun7394@uni.sydney.edu.au (J.J.); kben0192@uni.sydney.edu.au (K.B.B.); judy.kay@sydney.edu.au (J.K.); kalina.yacef@sydney.edu.au (K.Y.); irena.koprinska@sydney.edu.au (I.K.); 4 Heart Foundation of Australia, Sydney, NSW 2011, Australia; Kathy.Chapman@heartfoundation.org.au; 5 National Institute for Health Innovation, The University of Auckland, Auckland 1023, New Zealand; c.nimhurchu@auckland.ac.nz; 6 The George Institute for Global Health, Newtown, NSW 2042, Australia; 7 The University of New South Wales, Kensington, NSW 2052, Australia; 8 Prevention Research Centre, School of Public Health, The University of Sydney, Sydney, NSW 2006, Australia; adrian.bauman@sydney.edu.au

**Keywords:** food prepared outside the home, fast-food, energy-labelling, menu labelling, saturated fat, sugar, sodium, nutrition, young adults

## Abstract

Young adults are the highest consumers of food prepared outside home (FOH) and gain most weight among Australian adults. One strategy to address the obesogenic food environment is menu labelling legislation whereby outlets with >20 stores in one state and >50 Australia-wide must display energy content in kJ. The aim of this study was to assess the contribution of FOH to the energy and macronutrients, saturated fat, total sugars and sodium intakes of young Australians. One thousand and one 18 to 30-year-olds (57% female) residing in Australia’s most populous state recorded all foods and beverages consumed and the location of preparation for three consecutive days using a purpose-designed smartphone application. Group means for the daily consumption of energy, percentage energy (%E) for protein, carbohydrate, total sugars, total and saturated fats, and sodium density (mg/1000 kJ) and proportions of nutrients from FOH from menu labelling and independent outlets were compared. Overall, participants consumed 42.4% of their energy intake from FOH with other nutrients ranging from 39.8% (sugars) to 47.3% (sodium). Independent outlets not required to label menus, contributed a greater percentage of energy (23.6%) than menu labelling outlets (18.7%, *p* < 0.001). Public health policy responses such as public education campaigns, extended menu labelling, more detailed nutrition information and reformulation targets are suggested to facilitate healthier choices.

## 1. Introduction

During the transition from childhood to adulthood diet quality often declines [1]. As young adults leave the family home and begin to take responsibility for their own meals, many rely on meals and snacks prepared away from home. In Australia, young adults consume diets with a high energy density [2]; are the highest consumers of discretionary foods among adults [3]; and have lowest intakes of vegetables [4]. Young adults spend the largest proportion of their food budgets on eating out in Australia [5], the UK [6], European countries [7] and the US [8] compared with other age groups.

Many foods prepared outside the home (FOH) are energy-dense with high contents of nutrients that are of public health concern including saturated fat, sodium and added sugars [9,10]. Saturated fatty acids have been associated with an increased risk of stroke, and substitution of saturated fatty acids with mono- or poly-unsaturated fatty acids improves a range of biomarkers, including total cholesterol and glycaemic control [11]. Higher intakes of both total and added sugars are associated with increased risk of mortality from cardiovascular disease [12]. High sodium diets have detrimental effects on blood pressure, and adopting lower-sodium diets can significantly improve blood pressure in those with hypertension [13]. Diets high in these nutrients of public health concern are associated with chronic diseases that are responsible for about one third of all premature deaths in Australia [9].

Foods prepared outside the home include fast foods, other take-away foods, and café and restaurant meals and snacks. Regular consumption of fast food during young adulthood has been shown to be associated with weight gain and some metabolic abnormalities. In the US Coronary Artery Risk Development in Young Adults study, those who ate take-away food less than once per week gained 4.5 kg less over 15 years than regular consumers of two or more times per week [14]. Those who ate fast food two or more times per week also experienced a 104% greater increase in the homeostatic model assessment score, a measure of insulin resistance [14]. In an Australian cohort of women aged 26 to 36 years, those who consumed take-away food twice per week had significantly higher mean fasting glucose concentrations and the homeostatic model assessment score than those who did not [15].

There are limited recent Australian data concerning the contribution that FOH make to the diets of young adults. There are no data in the published scientific or publicly available literature on the usage of different types of food outlets and restaurants that are the main sources of these foods. Given that Australian young adults experience the largest weight gain per annum among adults [16], and the recognised deleterious effects of foods high in sodium, saturated fat and added sugar, determining the sources of meals and snacks is important. Across several Australian states, menu labelling for energy is currently mandated for food chains with more than 20 outlets in a single state or 50 across Australia, with similar approaches adopted by other countries [17]. However, the labelling does not extend to deleterious nutrients nor to smaller businesses such as independent restaurants, cafés and take-away shops as it is acknowledged that small businesses may not have the resources to have their menus evaluated. This study presented an opportunity to determine the contribution made to young adults’ dietary intakes by outlets included in and those outside the menu labelling legislation.

The aim of the Measuring Young adults’ Meals (MYMeals) study was to measure the proportions of total energy and nutrients of public health concern (sodium, saturated fat, and sugars) that food and beverages prepared outside home contribute to young adults’ diets and to assess the relative contributions that different food outlet types (i.e., fast food chains subject to menu labelling versus independent outlets) make to the overall food and beverage intake of young adults.

## 2. Materials and Methods

The MYMeals study was a cross-sectional survey of three-day dietary intake of 18 to 30-year-olds across Australia’s most populous state, New South Wales (NSW). The study protocol for MYMeals has been described in detail in a previous publication [18] with an outline of the procedures provided below. The study was approved by The University of Sydney Human Ethics Research Committee (project number 2016/546).

### 2.1. Sample Population

Participants were recruited from across NSW with purposive sampling to ensure the population included men and women, urban and rural dwellers from across the socioeconomic strata, and both 18- to 24-year and 25- to 30-year age groups. The selection of 18- to 30-years-old as the classification for young adulthood was to capture those who had attained legal age (18 years) and were in the process of becoming independent and transitioning to starting their own families. The planned sample size of 1008 participants provided a margin of error of ±3% on all estimates of proportions (with 95% confidence interval (CI)). Postcodes of each area were used to define geographic location as urban or rural/remote defined by the Accessibility/Remoteness Index of Australia [19] and socioeconomic status (SES) by the Socio-Economic Indexes for Areas [20].

### 2.2. Study Eligibility

To take part in the study, potential participants had to be within the age range specified (18 to 30-years-old); reside in NSW; eat at least one meal, snack or drink purchased outside the home each week; read and write English; and own or have access to a smartphone. Exclusion criteria were inability to commit to three consecutive days of dietary recording; pregnancy and/or breastfeeding; and a diagnosis of current or past eating disorder (for ethical reasons). Other medical conditions were not grounds for exclusion. However, participants were asked whether they were trying to lose, gain or maintain weight (see Section 2.5).

### 2.3. Recruitment Methods

Participants were recruited using a range of methods which included letters of invitation using names provided by the Australian Electoral Commission, electronic newsletters, public notice boards, social media via the two coordinating organisations (the University of Sydney and Cancer Council NSW), paid Facebook advertising, and the Relay for Life fundraising events held by Cancer Council NSW in urban and rural areas across the state. Snowball sampling, whereby young adults invited their peers, also occurred.

### 2.4. Procedures for Enrolment

Interested participants were directed to the study website to complete a screening survey for eligibility and to collect basic demographics and consent which was hosted on the REDCap research management system [21]. Demographic questions included sex (male, female or prefer not to say), age (under 18, 18–24 years, 25–30 years, or 31 years or older), education status (primary school or less, secondary school, trade qualification/apprenticeship/diploma or university degree), residential postcode to calculate SES (high or low) and geographic area (metropolitan or non-metropolitan). The system was programmed to allow researchers to assess progress in meeting quotas for all the designated demographic groups required with respect to age group, sex, SES and geographic location. Once a quota was filled e.g., 18 to 25-year-old females in the Greater Sydney area with a high SES, then no further participants were recruited to that group.

Upon enrollment, participants were contacted by the research team and allocated their dietary data collection dates and emailed instructions for download of the dietary assessment tool, the Eat and Track app [22], a user guide for the app and a copy of the Australian Bureau of Statistics Food Model booklet to estimate portion sizes [23]. The study website also had an electronic copy of the Food Model booklet and written instructions and videos on use of the app.

### 2.5. Assessment of Dietary Intake

The Eat and Track app was designed for recording of dietary intake for the MYMeals study and its usability and validation have been previously published [22,24,25]. Participants were allocated three specific consecutive days to record all food and beverages consumed, and across the population sample the start days were spread to capture weekdays and weekends. The app allowed participants to record meal occasions, including breakfast, lunch, dinner, and all snack and beverage consumption. The interface supported searches for common foods and commercial brand names, and had an extensive database of menu items from chain fast food restaurants and cafés that was developed from the Cancer Council NSW and The George Institute for Global Health’s fast food databases [26]. Other foods were derived from the database of the Australian Bureau of Statistics’ AUSNUT 2011–2013 [27,28,29]. In addition, the app supports freeform text entry of foods and beverages by the participants if they were unable to locate an item in the database of the app.

Participants recorded the serving size of each item consumed in grams or millilitres or selected a serving size from a predefined list based on portion sizes typically consumed by males and females aged 18 to 30 years in the most recent Australian national dietary survey [30]. For example, for foods such as pizzas, participants could choose to respond in grams, slices, or whole pizzas. Importantly the location from which the food was obtained was recorded. The app has a dropdown menu that included ‘home’ for all foods prepared inside the home and for locations outside the home the selection options were: bakery or patisserie; coffee chain; cold drinks chain; fast food chain; other take-away shop; ice cream parlour/frozen yoghurt; pub (public hotel) or club; service station or convenience store; and independent café or restaurant.

At the conclusion of each day’s recording, participants completed a short survey to ascertain if the eating behaviour was perceived to be typical or lower or higher than usual with respect to amount and frequency of foods prepared outside the home. Participants then completed a final demographics questionnaire after the three days of recording using the app. This collected self-reported height in centimetres and weight in kg, and 10% of the sample were measured by research staff to validate the measures [31]. Participants were asked to choose an option relating to their dieting behaviours i.e., “I am currently trying to lose weight”, “I am currently trying to stay the same weight”, “I am currently trying to gain weight” and “I am not trying to do anything about my weight”.

### 2.6. Data Cleaning

All the Eat and Track app food item entries were checked by research dietitians in the week following the data collection, and participants were contacted to clarify any manually entered freeform text entry food items, any obvious errors such as incorrect serving sizes, and missing meals. Manually entered foods (*n* = 5390, 18% of total entries) were matched to the nearest item in the food composition database of the app by one dietitian, and then checked by two others. Standard recipes in Taste Australia [32] were used for missing recipes. If the participant stated brand names for entered items that were not in the original database, Nutrition Information Panel data for that item were added to the database by the research dietitian (*n* = 1483, 5% of total entries). Two dietitians each checked all the data independently and any discrepancies were discussed until agreement was reached. All data were downloaded from the app including the location the food was prepared.

### 2.7. Statistical Analysis

The proportion of meal (a meal was recorded under one of the three meal button options i.e., breakfast, lunch, or dinner) or snack (recorded under the snack or beverage button) occasions prepared inside and outside of home were calculated. As participants were able to record multiple foods and beverages at each eating occasion, they could choose foods prepared from both inside the home and outside the home in the same meal, for example, when people ate breakfast at home but bought coffee from a café, or ate takeaway pizza at home but had a homemade cake for dessert. The number and proportions of meal occasions and snack and beverage occasions consisting of foods and beverages prepared inside the home, outside the home and a combination of inside and outside were calculated. Occasions of beverage consumption that consisted of drinking plain water only were excluded from this analysis. In addition, for eating occasions with food and beverages sourced outside home the frequency and proportions of meals, snacks and beverages consumed at outlets covered by menu labelling legislation and independent outlets, defined as those outlets not covered by menu labelling legislation, were investigated.

Daily energy and nutrient intakes for each participant were summed and the mean intakes for the three study days were calculated. Participants’ basal metabolic rates (BMR) were estimated using Schofield’s equation [33], based on the participants’ self-reported weight, age and sex as collected from the demographics questionnaire. Acceptable energy reporters were identified using Goldberg’s cut-offs for multiple-day dietary collection [34]. Any participants consuming an average energy intake (EI) over the three days of less than 1.0 × BMR were considered low energy reporters, and if they reported more than 2.4 × BMR they were deemed high energy reporters [35]. Results are presented for both total sample (*n* = 1001) and for acceptable energy reporters (EI:BMR = 1.0 to 2.4, *n* = 628).

Group means for the daily consumption of energy (kilojoules, kJ), and percentage energy (%E) for protein, carbohydrates, total sugars, total and saturated fats, and sodium density (mg/1000 kJ) for the three study days were calculated. The percentage of participants meeting the Acceptable Macronutrient Distribution Ranges (AMDR) [36], which are reference ranges set by the Australian National Health and Medical Research Council as appropriate intakes to balance intakes for healthy diets, was determined. The proportion of energy and nutrients from foods prepared at home and FOH were calculated. Sub-group analysis investigated differences in the proportion of energy and nutrients of foods eaten outside the home from chains covered by menu labelling and independent outlets using paired *t*-tests.

All data were analysed using IBM SPSS Statistics version 25 (IBM, SPSS Statistics, Armonk, NY, USA, 2017). Due to the multiple comparisons being conducted, the level of significance was reduced to *p* values ≤ 0.01.

## 3. Results

### 3.1. Participants

Overall, 1044 participants completed the study. Of these, four participants completed the study twice and were removed. Thirteen participants were excluded as they did not have three days of dietary intake data and another participant was excluded as they had collected three non-consecutive days. Five participants did not specify their gender and 20 (2%) participants either did not provide, or provided implausible, weight and/or height measurements. The final sample for analysis included 1001 participants. The days of data collection were spread for weekdays and weekends (57% Monday to Thursday and 43% Friday to Sunday). The characteristics of the included participants are shown in Table 1. The sample had a lower proportion of males than that of the Australian population (43% versus 49%), was more educated i.e., 68% with a tertiary qualification versus 56% for the general population [30], and 54% were in the top 50% for SES. As shown in Table 1, using EI:BMR for an individual based on three days, 36% of the sample (*n* = 364) were considered low energy reporters with nine participants considered high energy reporters. Of the low energy reporters, more than half (55%) reported that they were trying to lose weight.

### 3.2. Proportion of Meals and Snacks Prepared Away from Home

In total, there were 16,116 eating occasions (meals and snacks or drinks) during the study. Of the total eating occasions 5088 (31.6%) were entirely from FOH (see Table 2).

Of the recorded eating occasions, 8064 were meals (50% of total occasions), averaging eight meals per participant over three days (or 2.7 meals per day). Of the meals prepared outside the home, 920 (11% of total meals) were from outlets displaying menu labelling and 1410 meals (17% of total meals) were from independent outlets.

Additionally, there were 8052 snacks and drinks consumed during the study, averaging eight snack foods or beverages per participant over three days (or 2.7 snacks and drinks per day). Of the snacks and drinks prepared outside the home, 942 (12% of total snacks and drinks) were from menu labelling outlets and 1671 (21% of total snacks and drinks) were from independent outlets.

### 3.3. Average Daily Intakes of Energy and Nutrient Densities

The mean energy intake for the entire sample was 8050 kJ (SD 3063) per day (see Table 3). For the mean percentage energy intake from protein, 21% of participants reported intakes below the AMDR and 10% above the 25%E upper limit. For mean carbohydrate intakes (%E), 64% of participants were below the AMDR and only 1% above the upper limit. For the mean percentage energy from fat, only 1% of the sample was below the AMDR and 51% above the upper limit. When only acceptable energy reporters were included the mean total energy increased, protein %E, carbohydrate %E and total sugars %E declined modestly, and fat %E increased marginally.

### 3.4. Proportion of Nutrients Consumed from Food Prepared at Home and Food Prepared Outside the Home by Menu Labelling Status of the Food Outlet

Overall, participants consumed 42.4% of their average energy intake from FOH (Table 4). Proportional contribution of FOH to nutrient intakes was similar, ranging from 39.8% for total sugars to 47.3% for sodium being from FOH. Energy and nutrient contributions from independent food outlets were significantly higher than from chain outlets covered by menu labelling (all *p* < 0.001) for all nutrients.

The results for the 628 participants deemed to be ‘acceptable’ energy reporters on the basis of EI:BMR are shown in Table 5. The percentages of nutrients obtained from food prepared at home and FOH varied little from that observed when all participants were included in the analysis. In the case of food outlets with menu labelling versus independent outlets with no labelling, the relative contributions of energy and nutrients of public health concern from independent outlets were consistently greater (all *p* < 0.001).

## 4. Discussion

The present study provides the first comprehensive data about the contributions FOH make to the dietary intakes of young adult Australians. The primary finding of our study was that participants consumed about one-third of their meals and snacks and drinks from foods and beverages prepared outside of home and, of concern, these foods contributed more than 40% of total energy and nutrient intakes. Sodium contribution from FOH accounted for almost 47% of their total intake. A secondary finding was that independent outlets accounted for greater proportions of energy and all nutrients than did the outlets with menu labelling. This is of concern as independent outlets are not required to provide labelling of energy on their menus [17]. These findings demonstrate that the current energy menu labelling policy excludes the food outlets that make the largest contribution to nutritional intakes outside the home in this age group. The limited data available for NSW have demonstrated a 15% decrease in average kJ per meal after the introduction of menu labelling [37]. This supports the need to widen the scheme to capture more independent outlets. Furthermore, for those food outlets included within menu labelling regulations, provision of additional information about nutrients of public health concern such as sodium and saturated fat should be considered to provide them with additional information to guide their food selection.

More than half of the study participants were consuming intakes of fat and carbohydrates outside the recommended AMDR. Specifically, they were consuming a greater percentage of energy from fat and a smaller percentage from carbohydrate. The reasons for this are uncertain but the lower carbohydrate intakes are consistent with trends of a decline observed between the 1995 and 2011/12 National Nutrition Surveys [38], albeit, our current finding of 41.8%E in 2017/18 is lower than the 45.3%E reported in 2011/12 Survey for 19 to 30-year-olds [39]. The popular media has promoted a range of carbohydrate restricted diets for weight management such as low-carbohydrate, high-protein diets; the ketogenic diet; and the paleolithic diet, and whether this has influenced the young adults’ dietary patterns is an area for future investigation [40,41].

The total fat %E of 35.7 is higher than the 2011/12 National Nutrition Survey at 31.2%E [39]. While percentage energy from total fat may not be detrimental necessarily, the quality of fat must be considered. The present study found higher proportions of energy from saturated fat than is recommended. While the food prepared at home contributed almost 60% of saturated fat, FOH contributed more than 40% which is high when only one in three eating occasions was based on FOH. Earlier research from the 1995 Australian National Nutrition Survey found that 24% of saturated fat intake came from FOH [42]. The higher percentage found in the current study may reflect the greater household expenditure on eating out by Australian adults, and young adults spending more on FOH in this past decade [5]. Hence, the high saturated fat content of foods and beverages from both independent and menu labelling food chains is concerning. Improving the nutritional quality of food offerings in all outlets could result in favourable shifts in the consumption of saturated fat and researchers in the US have previously highlighted that concentrating only on fast food outlets and excluding independent outlets underestimated the exposure to FOH [43].

Perhaps of most concern is the contribution that FOH made to overall sodium intakes, almost half, while constituting about one third of the eating occasions. It is possible there may be some relative overestimation compared with meals consumed at home as added table salt was not always included in the app recording. However, table salt usually makes a relatively small contribution to total sodium intake compared with sodium within foods [44]. Reduction in population sodium intake is suggested to be one of the most cost-effective strategies for reducing premature death and disability attributable to high blood pressure and vascular disease [45]. While only a small sample, a survey of 338 adults across the age spectrum in Victoria reported how FOH at fast foods outlets, take-away outlets and restaurants contributed 28% of sodium intake [46]. The high sodium content of fast foods is well documented [47] and although the products of some leading fast-food chains have demonstrated some decreases in sodium content, it remains high [48]. An online experiment has reported the addition of sodium to menu labelling in addition to energy resulted in a decrease in the sodium content of the food items selected [49]. Research in the US suggests that individuals who are consciously trying to decrease their sodium intake decrease their frequency of eating meals FOH and particularly at fast food and pizza restaurants [50]. Thus, there would appear to be public support for substitution of menu items and reformulation of the high salt foods, labelling of sodium content and removal of added salt to FOH.

Comprehensive food reformulation can contribute to reductions in population intakes of nutrients of public health concern such as sodium and saturated fat [51]. In Australia, the Healthy Food Partnership, formerly the Australian Food and Health Dialogue, incorporates voluntary actions for reformulation but has had minimal success and slow progress, largely limited to sodium reduction in a limited selection of food categories [52,53].

The percentage energy from total sugars was within the recommended levels for total sugars (i.e., <20%) and less than that previously reported for the comparable age group in the 2011/12 National Nutrition and Physical Activity Survey for Australia which was 19.8%E from total sugars for 19 to 30-year-olds [39]. Our findings do not discriminate between added and total sugars as we did not have added sugar data in the database. Modelling has shown a reduction of intakes of total and added sugars has significant public health and economic benefits [54]. Limiting the availability and consumer choice of sugar-sweetened beverages and reformulating FOH are two potential efforts that could reduce the proportion of sugars consumed outside of home. Nutrition information that differentiates added sugars from total sugars is currently being considered for packaged foods in Australia [55] and should be considered as part of menu labelling.

The high proportion of energy intake from discretionary choices as well as the indication from focus groups of young adults in NSW that eating out is considered a ‘treat’ occasion means that there is a need for increased communication about discretionary choices and their role in the diet [56]. Education and information campaigns alone are not adequate to support healthy eating and must be underpinned by supportive and implemented structural policies including more comprehensive menu labelling, compositional limits for FOH, marketing restrictions for unhealthy foods and a health levy on sugar-sweetened beverages [51,56].

The current finding of 42% of total energy intake from FOH is comparable to findings in North America. Todd (2017) used the National Health and Nutrition examination Survey (NHANES) data collected between 2005 and 2014 [8]. It was shown that those born between 1981 to 1990 had the highest proportion of dietary energy from FOH; in the 2013/2014 survey this was 40% of total energy [8]. Similarly, the 2014 Canadian Community Healthy Survey reported a mean of 1972 calories were consumed from FOH versus 2038 calories from food at home for males 19 to 30-years-old [57]. For females, 1249 calories were from FOH and 1498 at home [57].

Strengths of the current study include sampling from across the state of NSW so that participants from both genders, with diverse socioeconomic status, from both urban and regional areas were included. However, potential differences by demographic characteristics are not presented here. The study was also the largest to date looking at eating out of home in Australia. In future, relative contributions of snacks versus meals will be explored. The app used to collect the dietary data was validated for nutrient densities [25]. It is acknowledged that estimation of accurate energy intakes is not possible using self-reporting measures. The high prevalence of low energy reporters (36%) is of concern but the nutrient densities i.e., percentage energy from macronutrients and sodium density, are less affected by under-reporting [58] as was demonstrated when we reported the findings with the low energy reporters removed. It must be acknowledged that 55% of the sample who were low energy reporters stated they were trying to lose weight, so intakes that were not considered plausible may in fact be so. A further limitation of the sample population is that it was a selection criterion for participation that at least one food or beverage consumed on a weekly basis was prepared outside the home and any individuals who very rarely or never consume foods other than FOH were excluded. Although participants needed to own a smartphone to participate, the current ownership for this age group is more than 97% and, therefore, this was not a limitation.

## 5. Conclusions

The MYMeals Study found that young adults in NSW consumed one-third of meals, snacks and drinks from FOH. However, FOH contributed more than 40% of energy, macronutrients, and nutrients of public health concern. These disproportionate amounts show the detrimental effects FOH have in the diets of young Australian adults. Independent outlets were making a greater contribution to intakes than the chain food outlets that must display menu energy-labelling, potentially limiting the reach of this initiative. Public health efforts to expand menu labelling to more outlets and the provision of more comprehensive nutrition information in tandem with healthier product replacements and reformulations alongside targeted health promotion to 18 to 30-year-olds are indicated. Such changes to food environments may support young adults to make healthier food choices but will need to be tested.

## Figures and Tables

**Table 1:** Sample demographic characteristics (*n* = 1001).

Participant Characteristics		*n* (%)
Gender	Female	566 (57)
	Male	435 (43)
Age group (years)	18–24	539 (54)
	25–30	462 (46)
Body Mass Index (mean kg/m^2^ (sd)) ^1^		25.1 (5.7)
Highest education attained	Secondary school or less	313 (31)
	Trade or diploma	165 (17)
	University degree	523 (52)
Socioeconomic status ^2^	High	584 (58)
	Low	398 (40)
Geographic location ^3^	Metropolitan	659 (66)
	Non-metropolitan	342 (34)
Dietary mis-reporting ^4^	Low energy reporters ^4^	364 (37)
	Trying to lose weight	200 (55)
	High energy reporters ^4^	9 (1)
Weight loss status	Trying to gain weight	2 (22)
	Trying to lose weight	446 (44)
	Trying to maintain weight	487 (49)
	Trying to gain weight	68 (7)

^1^ Mean and standard deviation. ^2^ From Socio-economic Indexes for Areas [20] based on residential postcode, lowest five deciles = low, highest five deciles = high; 19 postcodes could not be classified. ^3^ From Australian Statistical Geography Standard [19] Metropolitan was Sydney and all else became non-metropolitan. ^4^ Cut-off for Energy Intake: Basal Metabolic Rate (BMR) for an individual based on three days, low energy reporters 1.0 × BMR, high energy reporters BMR × 2.4 [35].

**Table 2:** Number and proportion of eating occasions sourced from foods from home and foods outside the home.

Location	Meals *n*	%	Snacks *n*	%	Total Occasions *n*	%
Home	4816	60	5439	68	10,255	63.6
Outside home	2475	31	2613	32	5088	31.6
Combination	773	9	-	-	773	4.8
Occasions outside of home only ^1^						
Menu labelling	920	11	942	12	1862	36.6
Independent	1410	17	1671	21	3081	60.6
Combination menu labelling and independent	145	2	-	-	145	2.8

^1^ Excluding meals from a combination of home and outside home.

**Table 3:** Consumption of energy (kJ) and nutrients (%E or mg/1000 kJ) as mean (SD) of three days intake (*n* = 1001).

Nutrient	Acceptable Macronutrient Distribution Range	Mean (SD) *n* = 1001	Mean (SD) for Acceptable Energy Reporters ^1^ *n* = 628
Energy kJ		8050 (3063)	9354 (2445)
Protein %E	15–25	19.0 (5.0)	18.2 (4.5)
Total fat %E	20–35	35.7 (7.2)	36.1 (7.1)
Saturated fat %E		12.5 (3.7)	12.9 (3.6)
Carbohydrate %E	45–65	41.8 (8.7)	39.3 (7.9)
Total Sugars %E		15.2 (6.4)	14.7 (5.7)
Sodium mg/1000 kJ		318 (109)	310 (107)

^1^ Acceptable energy reporting defined as an individual with Energy Intake:Basal Metabolic Rate <1.0 or >2.4 based on three days of intake [35].

**Table 4:** Mean (SE) ^1^ proportion of energy and nutrients contributed by foods prepared at home and outside of home and by menu labelling versus independent (non-menu labelling) food outlets (*n* = 1001).

Location	Energy %	Protein %	Total Fat %	Sat Fat ^2^ %	CHO ^3^ %	Total Sugars %	Sodium %
Home	57.6 (0.8)	58.5 (0.8)	56.8 (0.8)	57.0 (0.8)	57.3 (0.8)	60.2 (0.8)	52.7 (0.8)
Outside home	42.4 (0.8)	41.5 0.8)	43.2 (0.8)	43.0 (0.8)	42.7 (0.8)	39.8 (0.8)	47.3 (0.8)
Menu Labelling	18.7 (0.6)	17.4 (0.6)	18.9 (0.6)	19.0 (0.6)	20.3 (0.6)	19.4 (0.7)	21.9 (0.7)
Independent	23.6 (0.6)	24.1 (0.7)	24.2 (0.7)	24.2 (0.7)	22.4 (0.6)	20.4 (0.7)	25.3 (0.7)
*p* value	<0.001	<0.001	<0.001	<0.001	<0.001	<0.001	<0.001

^1^ SE = standard error. ^2^ Sat fat = saturated fat. ^3^ CHO = carbohydrate.

**Table 5:** Mean (SE) ^1^ proportion of energy and nutrients contributed by foods prepared at home and outside of home and by menu labelling versus independent (non-menu labelling) food outlets from ‘acceptable’ energy reporters only i.e., energy intake: basal metabolic rate, EI:BMR ≥ 1.0 and ≤2.4 (*n* = 628).

Location	Energy %	Protein %	Total Fat %	Sat Fat ^2^ %	CHO ^3^ %	Total Sugars %	Sodium %
Home	57.2 (0.9)	57.8 (1.0)	56.5 (1.0)	56.6 (1.0)	57.2 (0.9)	60.0 (1.0)	52.4 (1.0)
Outside home	42.8 (0.9)	42.2 (1.0)	43.5 (1.0)	43.4 (0.8)	42.8 (1.0)	40.0 (0.9)	47.6 (1.0)
Menu Labelling	18.1 (0.7)	16.8 (0.7)	18.0 (0.8)	17.9 (0.8)	19.6 (0.8)	18.4 (0.8)	21.1 (0.9)
Independent	24.7 (0.8)	25.4 (0.9)	25.5 (0.9)	25.5 (0.9)	23.2 (0.8)	21.6 (0.8)	26.5 (0.9)
*p* value	<0.001	<0.001	<0.001	<0.001	<0.001	<0.001	<0.001

^1^ SE = standard error. ^2^ Sat fat = saturated fat. ^3^ CHO = carbohydrate.

## Data Availability

Data sharing is not available for this study.
